# Individual characteristics associated with road traffic collisions and healthcare seeking in low- and middle-income countries and territories

**DOI:** 10.1371/journal.pgph.0002768

**Published:** 2024-01-19

**Authors:** Leila Ghalichi, Dina Goodman-Palmer, John Whitaker, Anne Abio, Michael Lowery Wilson, Lee Wallis, Bolormaa Norov, Krishna Kumar Aryal, Deborah Carvalho Malta, Till Bärnighausen, Pascal Geldsetzer, David Flood, Sebastian Vollmer, Michaela Theilmann, Justine Davies

**Affiliations:** 1 Institute of Applied Health Research, University of Birmingham, Birmingham, United Kingdom; 2 King’s Centre for Global Health and Health Partnerships, School of Life Course and Population Sciences, Faculty of Life Sciences and Medicine, King’s College London, London, United Kingdom; 3 Academic Department of Military Surgery and Trauma, Royal Centre for Defence Medicine, Birmingham, United Kingdom; 4 Injury Epidemiology and Prevention Research Group, Turku Brain Injury Centre, Division of Clinical Neurosciences, Turku University Hospital and University of Turku, Turku, Finland; 5 Research Centre for Child Psychiatry, University of Turku, Turku, Finland; 6 INVEST Research Flagship Center, University of Turku, Turku, Finland; 7 Clinical Services and Systems, Integrated Health Services, World Health Organization, Geneva, Switzerland; 8 Department of Nutrition and Food Safety, National Center for Public Health, Ulaanbaatar, Mongolia; 9 Bergen Centre for Ethics and Priority Setting, Department of Global Public Health and Primary Care, University of Bergen, Bergen, Norway; 10 Universidade Federal de Minas Gerais, Departamento de Enfermagem Materno Infantil e Saúde Pública, Belo Horizonte, MG, Brasil; 11 Institute of Global Health, Heidelberg University and University Hospital, Heidelberg, Germany; 12 Division of Primary Care and Population Health, Department of Medicine, Stanford University, Stanford, California, United States of America; 13 Chan Zuckerberg Biohub–San Francisco, San Francisco, California, United States of America; 14 Department of Medicine, University of Michigan, Ann Arbor, Michigan, United States of America; 15 Department of Economics and Centre for Modern Indian Studies, University of Göttingen, Göttingen, Germany; 16 Professorship of Behavioral Science for Disease Prevention and Health Care, Technical University of Munich, Munich, Germany; 17 Heidelberg Institute of Global Health, Heidelberg University, Heidelberg, Germany; 18 Centre for Global Surgery, Department of Global Health, Stellenbosch University, Cape Town, South Africa; SRMIST: SRM Institute of Science and Technology (Deemed to be University), INDIA

## Abstract

Incidence of road traffic collisions (RTCs), types of users involved, and healthcare requirement afterwards are essential information for efficient policy making. We analysed individual-level data from nationally representative surveys conducted in low- or middle-income countries (LMICs) between 2008–2019. We describe the weighted incidence of non-fatal RTC in the past 12 months, type of road user involved, and incidence of traffic injuries requiring medical attention. Multivariable logistic regressions were done to evaluate associated sociodemographic and economic characteristics, and alcohol use. Data were included from 90,790 individuals from 15 countries or territories. The non-fatal RTC incidence in participants aged 24–65 years was 5.2% (95% CI: 4.6–5.9), with significant differences dependent on country income status. Drivers, passengers, pedestrians and cyclists composed 37.2%, 40.3%, 11.3% and 11.2% of RTCs, respectively. The distribution of road user type varied with country income status, with divers increasing and cyclists decreasing with increasing country income status. Type of road users involved in RTCs also varied by the age and sex of the person involved, with a greater proportion of males than females involved as drivers, and a reverse pattern for pedestrians. In multivariable analysis, RTC incidence was associated with younger age, male sex, being single, and having achieved higher levels of education; there was no association with alcohol use. In a sensitivity analysis including respondents aged 18–64 years, results were similar, however, there was an association of RTC incidence with alcohol use. The incidence of injuries requiring medical attention was 1.8% (1.6–2.1). In multivariable analyses, requiring medical attention was associated with younger age, male sex, and higher wealth quintile. We found remarkable heterogeneity in RTC incidence, the type of road users involved, and the requirement for medical attention after injuries depending on country income status and socio-demographic characteristics. Targeted data-informed approaches are needed to prevent and manage RTCs.

## Introduction

Each year, about 1.35 million people die in road traffic collisions (RTCs) worldwide. RTCs are the leading cause of death among young people aged between 5 and 29 years [1]. In addition to fatalities, RTCs were responsible for 2.9% of all DALYs in 2019 (the seventh most important contributor) and were the main cause of DALYs for those aged 10–49 years [2]. But, despite their prevalence and consequences, RTCs have been neglected within global health discourse [3–5]. In an attempt to stop and reverse the increasing trend in RTC burden, the United Nations described 2011–2020 as the global Decade of Action for Road Safety. However, progress has been slow and the number of global road traffic deaths continues to increase [1]. The consequences of RTC-related injuries cause economic losses equivalent to approximately 3% of global GDP, rising to 5% in Low- and Middle-Income Countries (LMICs) [6, 7], making RTCs a substantial development issue.

Whilst the Millennium Development Goals had no targets related to injuries or RTCs, these receive more attention within the Sustainable Development Goal (SDG) agenda, with SDG 3.6 being to halve the number of global deaths and injuries due to RTCs by 2020 [8]. Unfortunately, despite RTCs being the leading cause of death for children and young adults aged 5–29 years, it has been largely ignored by the current child health agenda [1]. To increase global attention, the United Nations have now promoted a second global decade of Action for Road safety from 2021 to 2030 [9], with an extended–relative to the SDGs–target to reduce road traffic collision deaths by half by 2030 [10].

Nevertheless, from LMICs, there are little empirical data on the incidence of RTCs, the type of road user involved, the socio-economic and demographic characteristics of those involved, or the requirement for healthcare after RTCs. Whereas this information is essential to benchmark, develop efficient strategies to prevent injury, and ensure that healthcare is available to those who need it. Indeed, part of the explanation for why road safety remains low on the policy agendas of many LMICs may be that countries underestimate the scale of the problem [11, 12].

Insurance company databases, which could be used to understand the incidence, causes, and consequences of RTCs, are not available in many LMICs as many road users are not insured [13]. Many LMICs also lack the electronic health records necessary to record morbidity related to RTCs; only a handful of countries have trauma registries and even these are not nationwide [12, 14]. Therefore alternative data sources such as nationally representative surveys have been developed to better understand the scale, causes, and consequences of RTCs [12]. However, despite data being collected, such surveys are underused for informing important road safety metrics [12, 15]. The STEPS survey is a World Health Organisation instrument for Noncommunicable Disease Surveillance, Monitoring and Reporting. Violence and injury questions are included as an optional module [15, 16]. This tool, and others that are similar in methodology, exists in multiple major global languages and is conducted across multiple countries, offering a means for comparative analysis across countries. Whilst these surveys have allowed informative cross-national comparisons for NCDs, studies using them to inform cross-national understanding of incidence, morbidity outcomes, and factors associated with RTCs are lacking [17, 18].

In order to generate insights to inform preventative policy and strategies for management, using existing empirical data, we aimed to describe the incidence of non-fatal RTCs, the incidence of injuries requiring medical attention after RTCs, the type of road user involved RTCs, and explore socio-economic factors associated with RTCs and need for medical attention following RTCs.

## Methods

### Data sources

We analysed individual-participant-level data from nationally representative population-based surveys in LMICs extracted from the Global Health and Population Project on Access to Care for Cardiometabolic Diseases (HPACC) database. The search methods and requirements for survey inclusion into HPACC database have been described elsewhere [19]. The requirements for inclusion in this analysis were as follows: surveys had to 1) be conducted in the past 15 years (during or after 2008); 2) come from an upper-middle, lower-middle, or low-income country per World Bank definition during the survey year [20]; 3) be nationally representative; 4) have data available at the individual level; 5) include data on RTCs in the past 12 months; and 6) be harmonizable across age groupings. A summary of collation and cleaning methods and a brief description of included surveys is included in S1 Text. We included countries or territories and variables in the analyses if missingness was less than or equal to 15%.

### Outcome variables

Our main outcome variable was the incidence of non-fatal RTC, captured as an affirmative response to questions on involvement in any road traffic crash in the past 12 months. Our secondary outcome variables were first the type of road user involved in the RTC, namely whether they were cyclists (push bike), pedestrians, or road users of a motorized vehicle (and if so, if they were the driver or passenger), and second, the incidence of injuries requiring medical attention after RTC occurrence. Outcomes were derived from responses to questions on as detailed in S 1.

### Covariables: Socio-demographic, economic, and behavioural characteristics

Individual-level sociodemographic and economic variables used in the analysis are defined as follows. Age was captured in full years. Sex was captured as the binary variable, male or female. The following education categories were generated: no formal education or less than primary, completed primary school, some high school, and high school or above. Marital status was categorised as being married (married or cohabiting) or single (divorced, widowed or single). Area of residence was captured as urban or rural. Wealth was categorised in 5 quintiles based on harmonisation performed and described elsewhere [19]. In brief, if a survey included information on household assets, a principal component analysis was conducted to create household wealth quintiles. Otherwise, the quintiles are based on monthly household income. Countries and territories were classified by World Bank income status (low income, lower-middle income, upper-middle income) during the year of survey completion [20]. Recent alcohol consumption was categorized as a binary variable based on whether the respondent stated that they had consumed any alcohol in the previous 30 days.

### Statistical analyses

We used sampling weights to account for the survey specific sampling designs in all analyses. Survey weights were scaled so that each country or territory was weighted proportional to its total population size in 2015 [21]. More detail on the calculation of the sampling weights is described elsewhere [19]. All analyses were conducted using Stata 17.0.

In the primary descriptive analysis including data from all countries and territories, we describe the incidence of non-fatal RTCs in the age group 24–64 (where data were harmonizable for the maximum number of countries and territories) in the 12 months prior to the conduct of the survey. Incidence is described for the full sample, by World Bank income group, and by country or territory. For those who had a non-fatal RTC, the type of road user involved in the RTC is described for the entire sample, and by age, sex, education status, World Bank income group category, and country or territory. Results are reported as n and weighted %. The incidence of injury requiring medical attention after RTC is described. Univariable comparisons were done using Chi square.

To ascertain associations between sociodemographic factors with occurrence of RTC or injury requiring medical attention after RTC, we performed weighted logistic regressions. For occurrence of non-fatal RTC, regressions were run on the full sample, and for each country or territory. For occurrence of injury requiring medical attention after RTC, regressions were not run for individual countries or territories due to small sample size.

### Exploratory analyses

Alcohol use, household wealth, and household geographical location (rural or urban) have been shown to have important associations with prevalence of RTCs in other settings [22], so we included them in the RTC incidence regression models in addition to age, sex, marital status, and education. We included these variables when available for each survey (S2 Text).

### Sensitivity analyses

For the primary outcome, we repeated the main and exploratory analyses in the subset of countries or territories with data among 18–64 year olds, given that RTCs are known to be more prevalent in younger age groups [23].

### Ethics

The HPACC dataset was deemed “not human subjects research” by the institutional review board of the Harvard T.H. Chan School of Public Health in 2018 under protocol #IRB16-1915.

## Results

Surveys were available from 15 countries or territories, in all low or middle World Bank income categories, and with representation from 5 of 6 the WHO regions. Our analysis included two non-STEPs surveys (Brazil and Ghana) and 13 STEPS surveys (Algeria, Azerbaijan, Botswana, Eswatini, Georgia, Guyana, Kenya, Lesotho, Mongolia, Nepal, Rwanda, Timor Leste and Zanzibar) conducted between 2008 and 2019. Included countries and territories are shown in Table 1. No country was excluded due to missingness. In total, 90,790 patients aged 25–64 years took part in the surveys across all fifteen countries.

**Table 1:** Countries and their sample size used in the analyses.

	Survey name	World bank income category	WHO region	Number of participants (25–64)	Number of participants (18–64)
**Algeria**	STEPS	upper middle	African Region	5,732	6,537
**Azerbaijan**	STEPS	upper middle	European Region	2,304	2,561
**Botswana**	STEPS	upper middle	African Region	2,930	3,719
**Brazil**	PNS	upper middle	Region of the Americas	44,667	52,490
**Eswatini**	STEPS	Lower middle	African Region	2,177	2,849
**Georgia**	STEPS	Lower middle	European Region	3,189	3,486
**Ghana**	SAGE	Lower middle	African Region	3,069	---
**Guyana**	STEPS	upper middle	Region of the Americas	2,097	2,504
**Kenya**	STEPS	Lower middle	African Region	3,512	4,269
**Lesotho**	STEPS	Lower middle	African Region	2,284	---
**Mongolia**	STEPS	Lower middle	Western Pacific Region	4,361	5,266
**Nepal**	STEPS	Lower middle	South-East Asia Region	4,377	4,992
**Rwanda**	STEPS	low	African Region	5,654	6,744
**Timor Leste**	STEPS	Lower middle	South-East Asia Region	1,966	2,326
**Zanzibar**	STEPS	Lower middle	African Region	2,471	---

Table 1. shows the countries and number of participants included in the analysis.

*Ghana and Brazil were only included in the secondary analyses of requiring medical attention after an RTC.

The final pooled sample for the main analysis on non-fatal RTC incidence included 43,054 survey participants 24–65 years of age from 13 of the 15 countries (Brazil and Ghana were excluded from this due to only asking questions on the occurrence of injury after RTC). Females constituted 50.4% of the population and average age was 39.7 years (SD: 8.88). For the sensitivity analyses with participants aged 18–64, there were 45,253 survey participants from 11 countries; females constituted 50.0% of the population and average was 35.7 years (SD, 9.31). Characteristics of participants are shown in Tables 2 and 3 (Table B in S3 Text for participants used in the exploratory analyses).

**Table 2:** Characteristics of participants aged 25–64 included in the analyses of the primary outcome (RTC).

	All countries and participants aged 25–64	Participants aged 25–64 who suffered an RTC
**Number of participants**	43,054	1,664 (5.2% CI [4.6–5.9%])
**Number of countries**	13	13
**Income status**		
LIC (n, %)	8,125 (7.7%)	286 (6.5%)
L-MIC (n, %)	21,866 (56.4%)	754 (51.8%)
U-MIC (n, %)	13,063 (35.9%)	624 (41.7%)
**Age (mean, SD)**	39.7 (8.88)	37.8 (7.10)
**Sex**		
Male (n, %)	16,449 (49.5%)	1,043 (71.2%)
Female (n, %)	26,605 (50.5%)	621 (28.8%)
**Marital Status**		
Single	11,003 (22.4%)	475 (29.8%)
Not single	32,051 (77.6%)	1,189 (70.2%)
**Education**		
None or less than primary	14,374 (35.2%)	429 (26.2%)
Completed Primary	8,520 (21.0%)	383 (26.4%)
Some High School	8,890 (20.7%)	366 (20.1%)
High School or above	11,270 (23.1%)	486 (27.3%)

Results present actual numbers and weighted percentages.

**Table 3:** Characteristics of participants aged 25–64 included in the analyses for secondary outcome (requiring medical attention after injury).

	Participants who were asked about injury requiring medical attention after an RTCs	Participants who had an injury requiring medical attention after an RTC
**Number of participants**	74,534	1,292 (1.8% [CI 1.6–2.1%])
**Number of countries**	11	11
**Income status**		
LIC (n, %)	8,125 (5.7%)	132 (6.3%)
L-MIC (n, %)	17,342 (52.6%)	345 (60.4%)
U-MIC (n, %)	49,067 (41.7%)	815 (33.3%)
**Age (mean, SD)**	40.8 (10.05)	38.3 (9.08)
**Sex**		
Male (n, %)	31,143 (48.7%)	805 (73.1%)
Female (n, %)	43,391 (51.3%)	487 (26.9%)
**Marital Status**		
Single	32,449 (31.2%)	593 (32.7%)
Not single	42,085 (68.8%)	699 (67.3%)
**Education**		
None or less than primary	21,092 (31.7%)	266 (26.7%)
Completed Primary	19,214 (23.5%)	393 (28.7%)
Some High School	19,346 (21.5%)	379 (23.5%)
High School or above	14,882 (23.3%)	254 (21.1%)

Results present actual numbers and weighted percentages.

### Descriptive analyses of non-fatal RTC incidence and type of road user

**Fig 1:**
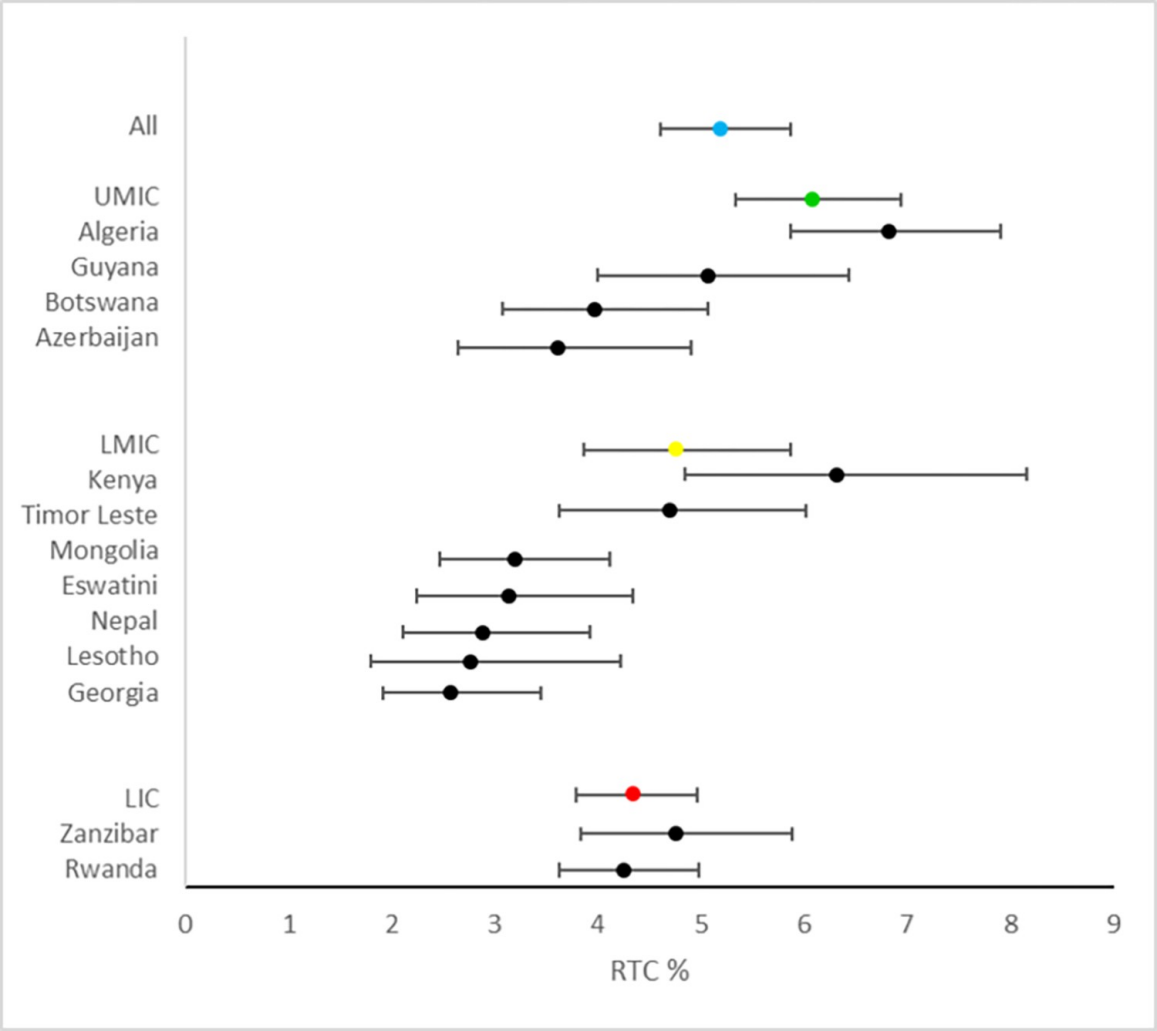
Incidence of RTC for individual countries by world bank income status. The average incidence for low-income countries is demonstrated in red, lower-middle income countries in yellow and higher middle-income countries are green. Horizontal bars represent 95% confidence intervals.

Across all countries and territories, incidence of non-fatal RTC in the previous 12 months was 5.2% (1,664/43,054) (Fig 1). There were significant differences in incidence of non-fatal RTC, dependent on country income status (p = 0.039); this was 4.3% (286/ 8,125) for low-income countries, 4.8% (754/21,866) for lower-middle income countries and 6.1% (624/13,063) for upper-middle income countries (Fig 1). Among people who suffered an RTC in the previous 12 months, 621/1,664 (29%) were female and the average age was 37.8 years (SD: 7.10) (Table 2).

**Fig 2:**
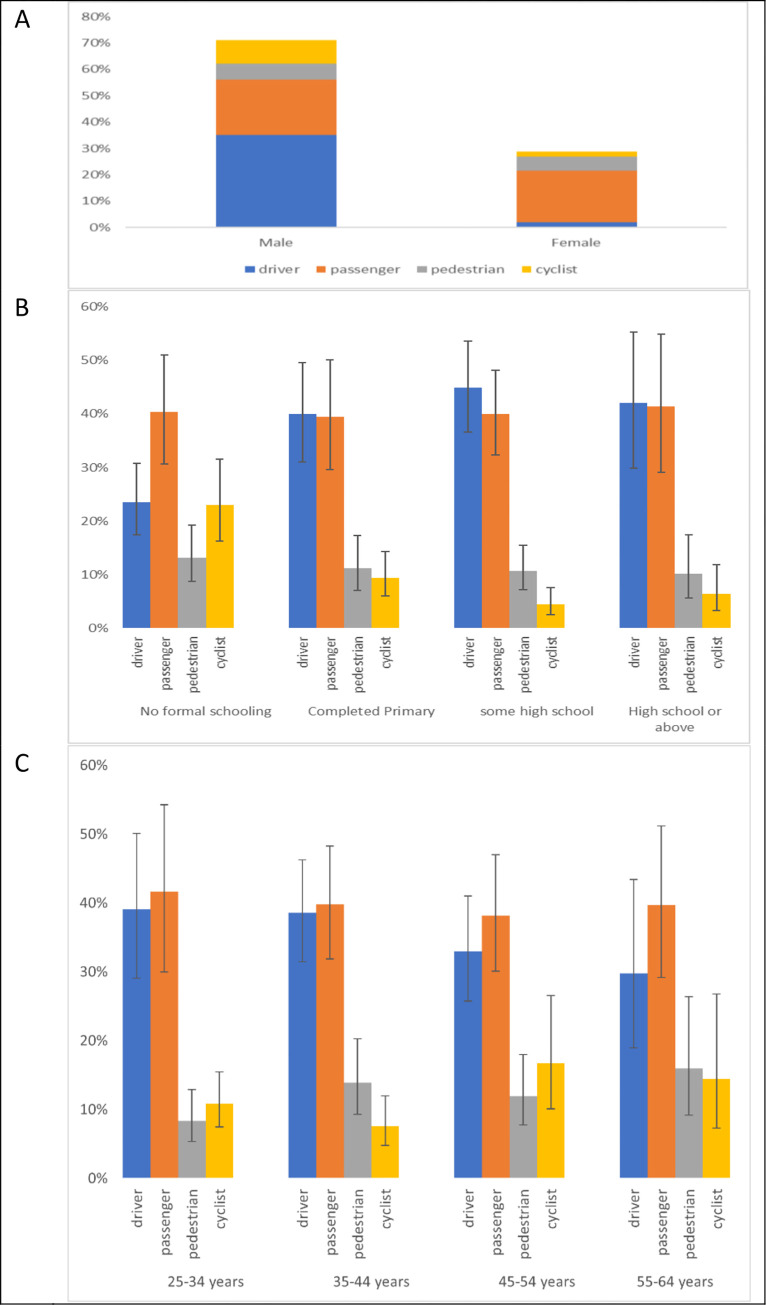
A–type of road user involved in RTC by sex in the age group 25–64 years. B. educational characteristics associated with RTC occurrence in the previous 12 months for age group 25–64 years. Vertical bars represent 95% confidence intervals. C. Age categories associated with RTC occurrence in the previous 12 months. Vertical bars represent 95% confidence intervals.

In thirteen countries or territories, including 43,356 participants (excluding Ghana and Brazil which did not ask about RTCs), participants were asked about the type of road user involved in the non-fatal RTC. Of the 1,664 respondents in these 13 countries and territories who had suffered an RTC in the past 12 months, 37.2% (608) were drivers, 40.3% (681) were passengers, 11.3% (181) were pedestrians, and 11.2% (194) were cyclists. A larger percentage of males who suffered an RCT were drivers (527/1,043 (49.4%)) than females (81/621 [7.2%]). In contrast, a larger percentage of females were involved as passengers (396/621 [67.4%]) than males (285/1,043 [29.4%]). The percentages of males involved as pedestrians (80/1,043 [8.4%]) was lower than females (101/621 [18.5%]) and among males, more were cyclists (12.8% [151/1,043]) compared with 6.9% (43/621) of females who were involved as cyclists (Fig 2A). Expanding the analysis to include individuals aged 18–24 did not change the results substantially (S4 Text).

Proportionately larger numbers of those with no formal education experienced an RTC as pedestrians or cyclists (47%) than those who had completed high school or higher (15%). Distribution of road user type by age categories did not vary substantially (Fig 2B and 2C).

Results of type of road users involved in non-fatal RTCs by World Bank income group and country or territory are reported in S5 Text. The proportion of RTCs involving cyclists decreased with increasing World Bank income group, whereas percentages of drivers increased.

### Associations with non-fatal RTC

In the main multi-variable analysis of non-fatal RTC incidence in participants aged 25–64 years, odds of suffering an RTC were lower in older people (0.99, 95%CI 0.98–1.00, p = 0.002), females (0.39, 95%CI: 0.33–0.46, p<0.001), and those who were married (0.70, 95%CI: 0.56–0.88, p = 0.002). There were greater odds of an RTC for those who had completed primary school (1.49, 95%CI: 1.19–1.88, p = 0.001), and for those who had completed secondary or more (1.32, 95%CI: 1.05–1.66, p = 0.017) compared to those with no education (Table 4). In the age range 18–64 years, the results are comparable to the main analysis involving participants aged 25–64 years old. Country-level results are reported in S6 Text.

**Table 4:** Multivariable analysis of the associations of having an RTC in the previous 12 months with age, sex, marital status, and education.

	Association of age, sex, marital, and education status on RTC in the previous 12 months (age category 25–64 years; 13 countries 43,054 participants)			Association of age, sex, marital, and education status on RTC in the previous 12 months (age category 18–64 years: 11 countries 45,253 participants)		
** **	OR	95% CI	P value	OR	95% CI	P value
**Age**	0.99	0.98–1.00	0.002	0.99	0.98–1.00	0.003
**Sex (Ref: Male)**	0.39	0.33–0.46	<0.001	0.39	0.34–0.46	<0.001
**Married or cohabiting (Ref: Single)**	0.70	0.56–0.88	0.002	0.79	0.65–0.95	0.013
**Education (Ref: no education or less than primary)**						
**Completed Primary**	1.49	1.19–1.88	0.001	1.40	1.10–1.78	0.007
**Some secondary**	1.13	0.87–1.47	0.357	1.02	0.80–1.30	0.875
**Completed secondary or more**	1.32	1.05–1.66	0.017	1.26	1.01–1.58	0.044

Surveys from 10 countries or territories with 32,476 participants had data available for age, sex, marital status, education status, and alcohol consumption in the past month (S7 Text). In this analysis, the associations of age, sex, and marital status with RTC were similar to those seen in the previous analysis that did not include alcohol consumption. There was no association between having consumed alcohol in the past month and incidence of RTC (OR: 1.13, 95%CI: 0.89–1.44, p = 0.330). However, for the sensitivity analysis conducted using data from eight countries with 36,879 participants aged 18–64 years, there was a positive association between consumption of alcohol in the past month and suffering an RTC (OR: 1.27, 95% CI: 1.00–1.61, p = 0.018).

Results for analyses showing associations including wealth and rural or urban residency are shown in S8 and S9 Texts. In particular, wealth and urban residence were associated with higher odds of suffering an RTC.

### Injuries requiring medical attention

Analyses of injuries requiring medical attention after RTC were done in survey participants from 11 countries between 24 and 65 years (from Algeria, Azerbaijan, Botswana, Brazil, Eswatini, Georgia, Ghana, Guyana, Kenya, Lesotho, Mongolia, Nepal, Rwanda, Timor Leste and Zanzibar; Algeria, Botswana, Georgia, and Mongolia did not ask questions on injuries requiring medical attention after RTC). From 74,534 respondents to the question for injuries requiring medical attention, 1292 (1.8%) had required medical attention after an RTC. Of these, 487 (27%) were female and the average age was 38.3 years (SD: 9.08). See Table 3 for characteristics of participants who suffered an RTC and required medical attention in the previous 12 months. The incidence of RTC requiring medical attention was 2.0% (132/ 8,125) for low-income countries, 2.1% (345/17,342) for lower-middle income countries and 1.5% (815/49,067) for upper-middle income countries, (P = 0.002).

**Fig 3:**
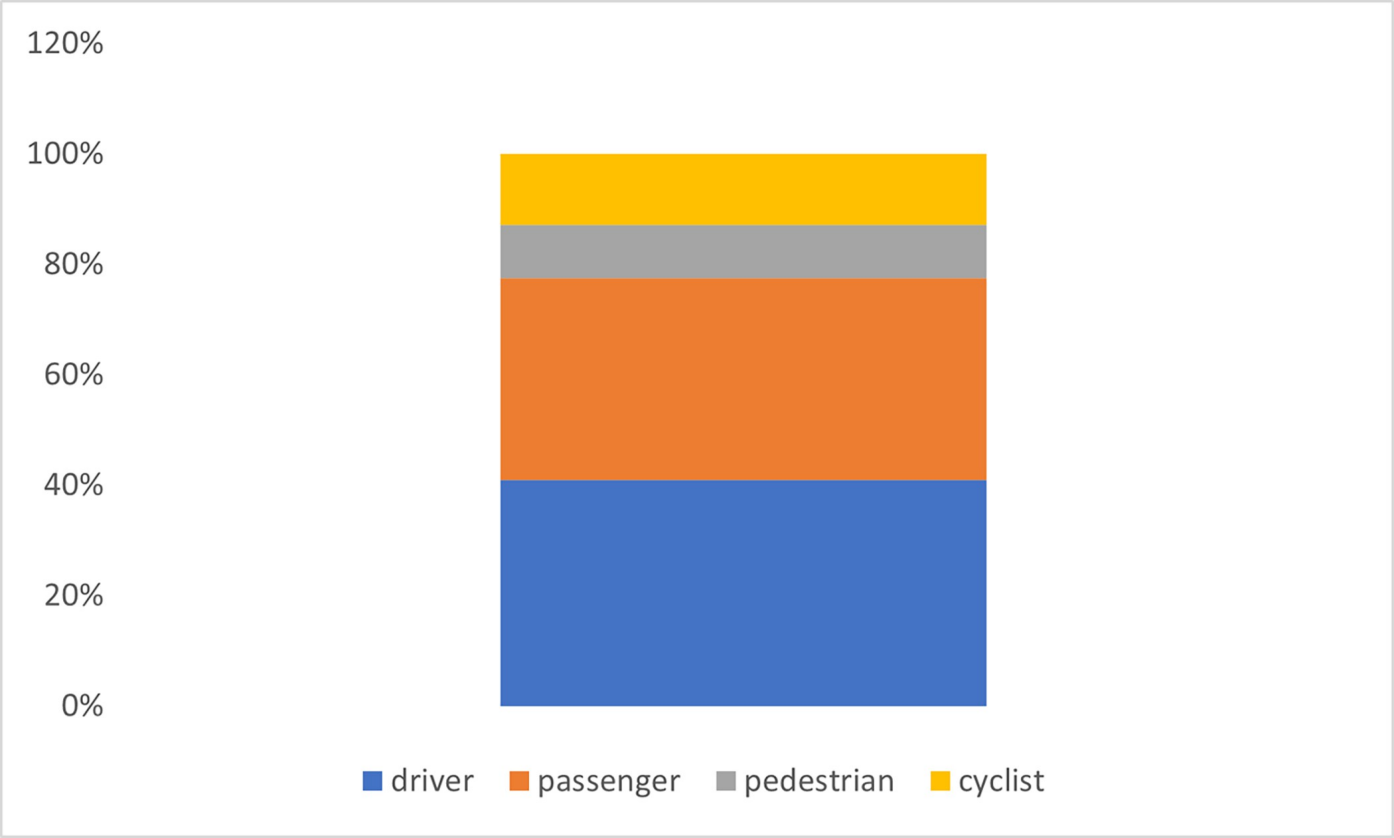
Types of road user requiring medical attention after experiencing an RTC.

In ten countries or territories (excluding Algeria, Botswana, Georgia, Ghana and Mongolia), 71,578 participants were asked about the type of road user involved in the RTC for which medical attention was required. Of the 1,256 respondents in these countries and territories who had suffered an RTC in the past 12 months and required medical attention, 41.0% (592) were drivers; 36.6% (408) were passengers; 9.6% (129) were pedestrians; and 12.8% (127) were cyclists (Fig 3). The proportion of cyclists requiring medical attention after an RTC was higher than other types of road users, although it was not statistically significant.

In the multi-variable analysis of associations requiring medical attention after RTC in participants aged 25–64 years living in 11 countries, odds were lower in older people (0.99, 95%CI 0.98–0.99, P value: <0.001), females (0.34, 95%CI: 0.26–0.44, P value<0.001), and not statistically different based on marital status or education (Table 5). In the three countries with data on wealth, we found a strong association between requiring medical attention after an RTC and increasing wealth quintile (S10 Text).

**Table 5:** Multivariable analysis of the associations of requiring medical attention after an RTC in the previous 12 months with age, sex, marital status, and education.

	Association of age, sex, marital, and education status on requiring medical attention after RTC in the previous 12 months (age category 25–64 years; 11 countries 74,534 participants)		
** **	OR	95% CI	P value
**Age**	0.98	0.97–0.99	<0.001
**Sex (Ref: Male)**	0.34	0.26–0.44	<0.001
**Married or cohabiting (Ref: Single)**	0.93	0.72–1.21	0.610
**Education (Ref: no education or less than primary)**			
**Completed Primary**	1.26	0.85–1.86	0.255
**Some secondary**	1.08	0.75–1.57	0.675
**Completed secondary or more**	0.88	0.57–1.35	0.557

## Discussion

Our study, which uses empirical individual respondent data from 15 nationally representative population-based surveys conducted in LMICs, found an estimated incidence of non-fatal RTCs of 5.2% in people aged 24–65 and 5.6% in people aged 18–64 years. There was a significant difference in incidence by country income group with incidence increasing with increasing country income group. We found positive associations of having suffered a non-fatal RTC with younger age, male sex, being single, and higher educational attainment. Most people who suffered an RTC were likely to be passengers, with drivers the second most common, ahead of pedestrians and cyclists. However, the type of road user affected differed by age, sex, and education status.

Whereas 5% of the study population suffered a non-fatal RTC, 2% of the population reported that they had an RTC which required medical attention, indicating a large proportion of those who had an RTC utilized their health system. However, this finding is likely to be an underestimate of the numbers who actually required care, as it relied on a self-reported measure which might reflect the numbers of people who actually sought healthcare after injury. It is likely that true morbidity after injury was greater than this. Issues in access to care after injury are related to healthcare availability and proximity and financial and cultural barriers, amongst others [24, 25]. Although other authors have used healthcare-seeking after injuries to indicate morbidity [26], we feel, for the reasons cited above, that this might be misleading and would not recommend using such estimates for healthcare planning. This difference highlights the need for country-specific data collection on occurrence of injuries after RTCs, as well as healthcare requirement. Simply relying on care seeking behaviour will likely underestimate the healthcare needs, particularly in less privileged areas and populations.

We found that odds of requiring medical attention was lower among older people and women. This could superficially indicate that older people and women are less severely injured than their younger/male counterparts. However, it might also be that females or older people are less able to seek healthcare; which may be due to cultural reasons [24, 25, 27].

Understanding the associations between socio-demographic characteristics and incidence of RTCs, including by types of road users, can assist with tailoring public health messaging or strategies to prevent injuries. For instance, we have found that types of road user involved in RTCs varies by country income level and sex and education status of those involved. This suggests that there might be a benefit to nuanced public health messaging to prevent RTCs. For example, we found that the proportion of cyclists involved in RTCs was greater in lower-income countries and among people with lower levels of education. Although cyclists were a relatively low proportion of those requiring healthcare after injuries in our study, a non-significantly higher proportion of them required medical attention after an RTC compared to other types of road users. This is in accordance to our other studies that showed a high mortality risk for cyclists [28]. Combined, these findings suggest that public health messaging and interventions around cycling safety may be useful in low income settings and for those with lower levels of education [29, 30].

The male sex predominance for road traffic collision observed in our study is in line with the pattern seen in other studies of RTCs specifically [26, 31] and injuries more generally [32–35]. This is consistent with the observed association that male sex has with multiple possible contributing factors including increased alcohol use, dangerous occupations, or higher risk taking behaviour [36, 37]. Our observation that more men who suffered RTCs were drivers compared to women could be a function of lower risk practices amongst women [31] or cultural norms leading to more men driving in the countries included in our study [38]. Differences in traffic collision risk could also be explained through occupational driving exposure and greater vehicle ownership [39]. Driving behaviour was not captured in our survey to aid this distinction, although we observed more males as drivers and more females as passengers which supports this hypothesis. Further, in studies where driving behaviour has been controlled for, a sex difference persists [38].

We also found a strong positive association between younger age and RTC, confirming another established aspect of burden from injuries in general and RTCs in particular [26, 31, 32, 40]. Some of this association is thought to relate to adolescent risk taking behaviours, alcohol use, disregard for speed limits, inadequate training and lack of experience [31, 37, 41, 42]. The negative association with RTC and being married in our study has been described in other studies [43] and supports a behavioural profile of lower risk taking being protective of sustaining an RTC [44, 45].

Interestingly, we found that alcohol consumption was not associated with RTCs in our main analysis but when younger age groups were included, there was an association, even though most of the population (around 80%) had not consumed alcohol in the past month. Our findings may be reflective of the greater risk-taking behaviour in younger populations [37, 46]. Impactful legislative changes to inhibit alcohol intake amongst drivers have been credited with driving down RTC rates in some countries [47] and the dose risk relationship between alcohol consumption and risk of RTC is well established [48]. Of the countries included in our analysis, all have regulations to prevent excessive alcohol use and driving [49]. The alcohol consumption variable used in our analysis asked about the preceding 30 days, and this would not necessarily correspond to alcohol intake around the time of a reported collision which may explain why the survey did not identify an association with alcohol at all age groups. But the signal association that we found for younger age supports targeting alcohol-free driving interventions at the younger adult age group.

Differences in access, use, and type of motorised transport might explain the observed association between incidence of RTC and lower educational attainment, which became insignificant when adding wealth to the model, likely because education and wealth have similar associations with transport use. That people with lower educational attainment who suffered RTCs were most likely to be pedestrians supports this hypothesis. Wealth and greater access to motorised transport also likely explains our observed transition from bicycle to motorised transport RTCs at the population level from lower to middle income settings [50].

We found a strong association between rural and urban living status and RTC incidence, with odds of RTCs being lower in rural than urban dwellers. This may be due to lower traffic density or speeds achievable in rural areas. However, we didn’t find any difference in the proportions of road user types who suffered a non-fatal RTC by geographical location (Fig C in S5 Text). Other single-site studies have also noted difference in RTC rates between urban and rural settings [33, 34]. Combined, the evidence suggests a greater need to target road safety measures in urban areas, although no need for differentiation in messaging or strategy by type of road user in different areas.

Our study has some limitations. We have only included adults aged over 25 in our main analysis, although our analysis with participants over 18 years did include more individuals, even if from fewer countries. Due to data constraints, we excluded children, which for the many LMICs with young population profiles could mean that as much as half the population were not included [51]. Although the Global Schools Based Health Survey collects data on occurrence and causes of accidents in adolescents, data from younger children is sparce [37]. Given the low number of respondents to the question regarding type of road users involved in an accident, we were likely underpowered for detecting differences in requiring medical attention.

We have been able to describe the characteristics of those involved in a road traffic collision, however, since we have no information such as type of vehicle use for those not involved in a collision, we are unable to perform comparative analyses to draw clearer implications for preventative policy intervention. We also do not have data on fatalities after RTCs, therefore survivorship bias may be affecting our results. Among countries and territories participating in this study, Brazil has a much larger population than the other countries and thus might dominate the results. Similarly, only around 5% of survey participants were from LICs which would limit the survey power to detect differences between the income strata and limits the generalisability of our findings to this population.

Not all countries included all relevant indicators in their surveys meaning our analyses of the variables alcohol use, household wealth, and household residency were exploratory only. Not all STEPS survey participants were asked a consistent selection of questions. This can inhibit attempts to conduct large cross national comparative analyses. However, a greater barrier to a wider spread use of the STEPS tool to inform injury epidemiology and intervention is the lack of inclusion of the injury question set within all national surveys. Out of 72 country surveys made available to this research collaborative only 15 included the injury question sets. More advocacy is needed from the injury research community to emphasise the important and often neglected disease burden warranting inclusion of related survey collection tools.

## Conclusion

Whereas previous studies describing RTCs have generally used data from single countries or modelled data. Our study, uniquely, uses empirical data collected using similar methodologies from individual participants in nationally representative samples of different countries. We found remarkable heterogeneity in epidemiology of RTC and its association with population characteristics between countries, despite similarities in their socioeconomic characteristics and population structure. These findings should help to inform tailored injury prevention strategies and start to inform health care strategies which match need in LMICs.

## Supporting information

S1 Text Summary of survey collation and cleaning methods and included surveys and RTC questions. (DOCX)

S2 Text Summary of countries included in the main and exploratory analyses for the age categories 25–64 (Table 2A) and 18–64 years (Table 2B). (DOCX)

S3 Text Characteristics of participants used in the main analysis and each exploratory analysis for the 25–64-year age group (Table 2A) and the 18–64-year age group (Table 2B). (DOCX)

S4 Text Types of road users involved in RTCs in the age group 18–64. (DOCX)

S5 Text Percentages of each type of road user involved in an RTC by World Bank income status, country and geographical location. (DOCX)

S6 Text Associations with occurrence of a non-fatal RTC in the previous 12 months for individual countries. (DOCX)

S7 Text Results of binary logistic analyses ascertaining the associations with non-fatal RTC and age, sex, marital and education status, and alcohol use in the past month in participants aged 25–64 years and 18–64 years. (DOCX)

S8 Text Results of binary logistic analyses ascertaining the associations of age, sex, marital and education status, and wealth in participants aged 25–64 years and 18–64 years. (DOCX)

S9 Text Associations of rural or urban habitation on suffering an RTC. (DOCX)

S10 Text Multivariable analysis of the associations of requiring medical attention after an RTC in the previous 12 months with age, sex, marital status, and wealth. (DOCX)
