# Supplementary material for: Individual characteristics associated with road traffic collisions and healthcare seeking in low- and middle-income countries and territories
Source: PLOS Glob Public Health. 2024 Jan 19;4(1):e0002768. doi: 10.1371/journal.pgph.0002768 (PMC10798533; doi:10.1371/journal.pgph.0002768)
Supplement: S1 Text — (DOCX) [file pgph.0002768.s001.docx]

**S1**

Summary of survey collation and cleaning methods and included surveys and RTC questions:

Data used in this analysis was extracted mainly from STEPS survey with optional module of “violence and injury”. Other surveys used were PNS (in Brazil) and SAGE (in Ghana). Methods and questionnaires used in each of these surveys can be found at the below sources:

# STEPs: <https://www.who.int/ncds/surveillance/steps/instrument/en/>

# SAGE: <https://apps.who.int/healthinfo/systems/surveydata/index.php/catalog/6>

# PNS, Brazil: [https: //www.ibge.gov.br/en/statistics/social/justice-and-security/16840-national-survey-of-health.html?edicao=19375&t=sobre](https://www.ibge.gov.br/en/statistics/social/justice-and-security/16840-national-survey-of-health.html?edicao=19375&t=sobre)

|  | **Survey name** | **Year of survey** | **Occurrence of RTC in the past 12 month** | | **Type of road user involved in the RTC** | **Being involved in an RTC in the past 12 months which required medical attention** | **Type of road users** |
| --- | --- | --- | --- | --- | --- | --- | --- |
| **Algeria** | STEPS | 2016 | × | | × | N/A | × |
| **Azerbaijan *** | STEPS | 2017 | × | | × | × | × |
| **Botswana** | STEPS | 2014 | × | | × | N/A | × |
| **Brazil** | PNS | 2013 | N/A | | N/A | × | × |
| **Eswatini** | STEPS | 2014 | × | | × | × | × |
| **Georgia** | STEPS | 2016 | × | | × | N/A | × |
| **Ghana** | SAGE | 2007-2008 | | N/A | N/A | × | N/A |
| **Guyana *** | STEPS | 2016 | × | | × | × | × |
| **Kenya** | STEPS | 2015 | × | | × | × | × |
| **Lesotho** | STEPS | 2012 | × | | × | × | × |
| **Mongolia** | STEPS | 2013 | × | | × | N/A | × |
| **Nepal** | STEPS | 2019 | × | | × | × | × |
| **Rwanda** | STEPS | 2012 | × | | × | × | × |
| **Timor L’este** | STEPS | 2014 | × | | × | × | × |
| **Zanzibar** | STEPS | 2011 | × | | × | × | × |

PNS, Pesquisa Nacional de Saúde; SAGE, Study on global AGEing and adult health; STEPS, WHO STEPwise approach to surveillance;

*****. Azerbaijan and Guyana did not include cyclists in their road type user question.

The main outcome questions:

STEPS: In the past 12 months, have you been involved in a road traffic crash as a driver, passenger, pedestrian, or cyclist?

Secondary outcome 1:

STEPS: In the past 12 months, have you been involved in a road traffic crash as a driver, passenger, pedestrian, or cyclist?

PNS: During the most severe accident in the past 12 months, were you Driver of a car/van, Bus driver, Truck driver, Motorbike rider, Bicycle rider, Passenger in a, car/van, Passenger in a bus, Passenger in a truck, Passenger on a motorbike, Passenger on a bicycle, Pedestrian or other. (The answers were later recoded into four categories of Driver, Passenger, Pedestrian and Cyclist)

Secondary outcome 2:

STEPS: Did you have any injuries in this road traffic crash which required medical attention?

SAGE: Did you receive any medical care or treatment for your injuries?

PNS: Did you receive medical treatment because of this accident?
