# Supplementary material for: Individual characteristics associated with road traffic collisions and healthcare seeking in low- and middle-income countries and territories
Source: PLOS Glob Public Health. 2024 Jan 19;4(1):e0002768. doi: 10.1371/journal.pgph.0002768 (PMC10798533; doi:10.1371/journal.pgph.0002768)
Supplement: S2 Text — (DOCX) [file pgph.0002768.s002.docx]

**S2**

Summary of countries included in the main and exploratory analyses for the age categories 25-64 (table 2a) and 18-64 years (table 2b)

Appendix Table 2a.

|  | **Survey name** | **Number** | **Main analysis** | **Alcohol consumption in the past 30 days** | **Wealth quintile** | **Household residency** |
| --- | --- | --- | --- | --- | --- | --- |
| **Algeria** | STEPS | 5,746 | × | × |  | × |
| **Azerbaijan** | STEPS | 2,340 | × | × |  | × |
| **Botswana** | STEPS | 2,934 | × | × |  |  |
| **Brazil** | PNS | 44,659 |  |  |  |  |
| **Eswatini** | STEPS | 2,181 | × |  |  |  |
| **Georgia** | STEPS | 3,392 | × |  | × | × |
| **Ghana** | SAGE | 3,069 |  |  |  |  |
| **Guyana** | STEPS | 2,098 | × |  |  |  |
| **Kenya** | STEPS | 3,513 | × | × | × | × |
| **Lesotho** | STEPS | 2,284 | × | × | × |  |
| **Mongolia** | STEPS | 4,362 | × | × | × | × |
| **Nepal** | STEPS | 4,378 | × | × |  | × |
| **Rwanda** | STEPS | 5,660 | × | × |  |  |
| **Timor L’este** | STEPS | 1,997 | × | × |  |  |
| **Zanzibar** | STEPS | 2,471 | × | × | × | × |

Appendix table 1a. shows the countries and number of participants included in the study for the main analysis (age 25-64) and the countries used in the main and exploratory binary logistic regression analyses. PNS is Pesquisa Nacional de Saúde; SAGE is Study on Global AGEing and Adult Health

Appendix Table 2b.

|  | **Number** | **Main analysis** | **Alcohol consumption in the past 30 days** | **Wealth quintile** | **Household residency** |
| --- | --- | --- | --- | --- | --- |
| **Algeria** | 6,654 | × | × |  | × |
| **Azerbaijan** | 2,608 | × | × |  | × |
| **Botswana** | 3,788 | × | × |  |  |
| **Brazil** | 56,237 |  |  |  |  |
| **Eswatini** | 3,081 | × |  |  |  |
| **Georgia** | 3,699 | × |  | × | × |
| **Ghana** | 3,135 |  |  |  |  |
| **Guyana** | 2,509 | × |  |  |  |
| **Kenya** | 4,282 | × | × | × | × |
| **Lesotho** | 2,310 |  |  |  |  |
| **Mongolia** | 5,268 | × | × | × | × |
| **Nepal** | 5,060 | × | × |  | × |
| **Rwanda** | 6,812 | × | × |  |  |
| **Timor L’este** | 2,407 | × | × |  |  |
| **Zanzibar** | 2,488 |  |  |  |  |

Appendix table 2b shows the countries and number of participants aged 18-64 included in the study for the main analysis and the countries used in the main and exploratory binary logistic regression analyses.
