# Supplementary material for: Individual characteristics associated with road traffic collisions and healthcare seeking in low- and middle-income countries and territories
Source: PLOS Glob Public Health. 2024 Jan 19;4(1):e0002768. doi: 10.1371/journal.pgph.0002768 (PMC10798533; doi:10.1371/journal.pgph.0002768)
Supplement: S3 Text — (DOCX) [file pgph.0002768.s003.docx]

**S3**

Characteristics of participants used in the main analysis and each exploratory analysis for the 25–64-year age group (table 2a) and the 18–64-year age group (table 2b)

S3 Table A**. Characteristics of participants in the exploratory analyses: 25–64-year-olds**

|  | **Including alcohol consumption in the past 30 days** | **Including wealth quintile** | **Including rural or urban residence** |
| --- | --- | --- | --- |
|  |  |  |  |
| **Number of participants *** | 32,476 | 15,042 | 25,946 |
| **RTC incidence% 95% CI** | 5.1 | 5.7 | 5.4 |
|  | 4.6 – 5.8 | 4.5 – 7.2 | 4.7 – 6.1 |
| **Income status** |  |  |  |
| LIC (n, %) | 7,479 (8.1%) | 2,292 (3.4%) | 2,471 (1.6%) |
| L-MIC (n, %) | 14,936 (54.5%) | 12,750 (96.6%) | 15,439 (60.1%) |
| U-MIC (n, %) | 10,061 (37.4%) | - | 8,036 (38.3%) |
| **Age: mean, (SD)** | 39.7 | 38.8 | 39.8 |
|  | (8.17) | (8.35) | (7.34) |
| **Sex** |  |  |  |
| Male (n, %) | 12,434 (47.5%) | 5,752 (49.9%) | 10,285 (49.8%) |
| Female (n, %) | 20,042 (52.5%) | 9,290 (50.1%) | 15,661 (50.2%) |
| **Marital Status** |  |  |  |
| Single | 7,736 (22.3%) | 3,352 (24.7%) | 4,872 (21.2%) |
| Not single | 24,740 (77.7%) | 11,690 (75.3%) | 21,074 (78.8%) |
| **Education** |  |  |  |
| None or less than primary | 12,352 (36.7%) | 3,726 (35.1%) | 7,174 (33.3%) |
| Completed Primary | 6,341 (20.9%) | 2,400 (21.7%) | 3,931 (20.4%) |
| Some High School | 6,630 (21.3%) | 2,307 (9.9%) | 5,841 (21.6%) |
| High School or above | 7,153 (21.1%) | 6,609 (33.3%) | 9,000 (24.7%) |
| No | 24,638 (79.6%) |  |  |
| Yes | 7,838 (20.4%) |  |  |
| **Wealth Quintiles** |  |  |  |
| Q1 |  | 3,254 (19.6%) |  |
| Q2 |  | 3,232 (20.3%) |  |
| Q3 |  | 3,161 (18.8%) |  |
| Q4 |  | 2,830 (19.7%) |  |
| Q5 |  | 2,565 (21.6%) |  |
| **Geography** |  |  |  |
| Urban (n, %) |  |  | 11,992 (42.6%) |
| Rural (n, %) |  |  | 13,954 (57.4%) |

*Numbers in the multivariable analyses are lower than those used in the main descriptive analyses, given missingness of some variables.

Appendix table 3a Characteristics of participants aged 25-64 included in the main and exploratory analyses. Results present actual numbers and weighted percentages.

S3 Table B. **Characteristics of participants in the sensitivity analysis: 18–64-year-olds**

|  | **All countries and participants for main RTC analysis** | **Participants aged 18-64 who suffered and RTC** | **Exploratory analysis 1: Including alcohol consumption in the past 30 days** | **Exploratory analysis 2: Including wealth quintile** | **Exploratory analysis 3: Including rural or urban residence** |
| --- | --- | --- | --- | --- | --- |
| **Number of participants** | 45,253 | 1910 | 33,292 | 12,412 | 27,111 |
| **RTC incidence% 95% CI** | 5.6 | N/A | 5.6 | 5.7 | 5.7 |
|  | 5.1 – 6.3 |  | 5.0 – 6.2 | 4.6 – 7.1 | 5.1 – 6.4 |
| **Income status** |  |  |  |  |  |
| LIC (n, %) | 6,744 (7.4%) | 275 (6.9%) | 6,124 (7.7%) | - | - |
| L-MIC (n, %) | 23,188 (57.0%) | 874 (50.6%) | 15,366 (55.0%) | 12,412 (100.0%) | 18,013 (62.3%) |
| U-MIC (n, %) | 15,321 (35.6%) | 761 (42.5%) | 11,802 (37.3%) | - | 9,098 (37.7%) |
|  |  |  |  |  |  |
| **Age (mean, SD)** | 35.7 | 33.4 | 35.4 | 34.8 | 35.9 |
|  | 9.31 | 7.45 | 8.42 | 7.79 | 7.64 |
| **Sex** |  |  |  |  |  |
| Male (n, %) | 17,425 (50.0%) | 1,198 (71.4%) | 12,871 (48.1%) | 4,884 (50.5%) | 10,785 (50.1%) |
| Female (n, %) | 27,828 (50.0%) |  | 22,421 (51.9%) | 7,528 (49.5%) | 16,326 (49.9%) |
|  |  | 712 (28.6%) |  |  |  |
|  |  |  |  |  |  |
| **Marital Status** |  |  |  |  |  |
| Single | 14,425 (32.5%) | 730 (41.9%) | 9,997 (32.7%) | 3,199 (33.6%) | 6,656 (31.1%) |
|  |  |  |  |  |  |
| Not single | 30,828 (67.5%) | 1,180 (58.1%) | 23,295 (67.3%) | 9,213 (66.4%) | 20,455 (68.9%) |
|  |  |  |  |  |  |
| **Education** |  |  |  |  |  |
| None or less than primary | 13,315 (31.1%) | 446 (23.6%) | 11,263 (32.3%) | 1,903 (31.3%) | 6,373 (29.2%) |
|  |  |  |  |  |  |
| Completed Primary | 8,402 (20.7%) | 403 (25.3%) | 5,989 (20.5%) | 1,307 (20.2%) | 3,900 (20.3%) |
|  |  |  |  |  |  |
| Some High School | 10,092 (23.2%) | 441 (22.0%) | 7,397 (23.9%) | 1,734 (11.3%) | 6,204 (23.7%) |
|  |  |  |  |  |  |
| High School or above | 13,444 (25.0%) | 620 (29.1%) | 8,643 (23.3%) | 7,468 (37.2%) | 10,634 (26.8%) |
|  |  |  |  |  |  |
| **Alcohol in last month** |  |  |  |  |  |
| No |  |  | 25,146 (81.1%) |  |  |
|  |  |  |  |  |  |
| Yes |  |  | 8,146 (18.9%) |  |  |
|  |  |  |  |  |  |
| **Wealth Quintile** |  |  |  |  |  |
| Q1 |  |  |  | 2,466 (18.8%) |  |
|  |  |  |  |  |  |
| Q2 |  |  |  | 2,578 (20.0%) |  |
|  |  |  |  |  |  |
| Q3 |  |  |  | 2,666 (18.2%) |  |
|  |  |  |  |  |  |
| Q4 |  |  |  | 2,572 (20.1%) |  |
|  |  |  |  |  |  |
| Q5 |  |  |  | 2,130 (22.9%) |  |
|  |  |  |  |  |  |
| **Geography** |  |  |  |  |  |
| Urban (n, %) |  |  |  |  | 13,062 (42.9%) |
|  |  |  |  |  |  |
| Rural (n, %) |  |  |  |  | 14,049 (57.1%) |
|  |  |  |  |  |  |

*Numbers in the multivariable analyses are lower than those used in the main descriptive analyses, given missingness of some variables.

Appendix Table 3b Baseline characteristics of all participants included in the sensitivity analyses aged 18-64 (complete and exploratory datasets). Results present actual numbers and weighted percentages.
