# Supplementary material for: Individual characteristics associated with road traffic collisions and healthcare seeking in low- and middle-income countries and territories
Source: PLOS Glob Public Health. 2024 Jan 19;4(1):e0002768. doi: 10.1371/journal.pgph.0002768 (PMC10798533; doi:10.1371/journal.pgph.0002768)
Supplement: S4 Text — (DOCX) [file pgph.0002768.s004.docx]

**S4**

Types of road users involved in RTCs in the age group 18-64

Among 11 countries, including 46,168 participants, asked about the type of road user involved in the RTC in the age group 18-64. Of the 1,918 respondents who had suffered an RTC in the past 12 months, 35% (673) were drivers; 39% (784) were passengers; 12% (210) were pedestrians; and 14% (251) were cyclists.

Appendix Figure 4. Shows RTC type in the population 25-64 years and 18-64 years.

Among the 1918 respondents who suffered an RTC aged 18-64 years old, a larger percentage of males were drivers (582/1,201 (45%)) than was seen in females who suffered an RTC (91/717 (9%)), whereas a larger percentage of females were involved as passengers (447/717 (64%)) than males (337/1,201 (30%)). The percentages of males involved as pedestrians (90/1,201 (9%)) was lower than seen for females who were involved in an accident (120/717 (19%)) and as cyclists was higher - (16% (192/1,201) of males were involved as cyclists compared with 8% (59/717) of females).

Among those who suffered an RTC aged 18-64 years old, the type of road user involved in accidents was affected by educational status, with proportionately larger numbers of those with no formal education being injured as pedestrians or cyclists (totalling 41% of people injured and who had no formal education) compared with those who were injured and had complete high school or higher, where 19% of those injured were pedestrians or cyclists.
