# Supplementary material for: Individual characteristics associated with road traffic collisions and healthcare seeking in low- and middle-income countries and territories
Source: PLOS Glob Public Health. 2024 Jan 19;4(1):e0002768. doi: 10.1371/journal.pgph.0002768 (PMC10798533; doi:10.1371/journal.pgph.0002768)
Supplement: S5 Text — (DOCX) [file pgph.0002768.s005.docx]

**S5**

Percentages of each type of road user involved in an RTC by World Bank income status, country and geographical location.

In low-income countries, 32% of those who had accidents were cyclists, while this rate was 14% and 4% in Lower middle and upper middle income countries, respectively.


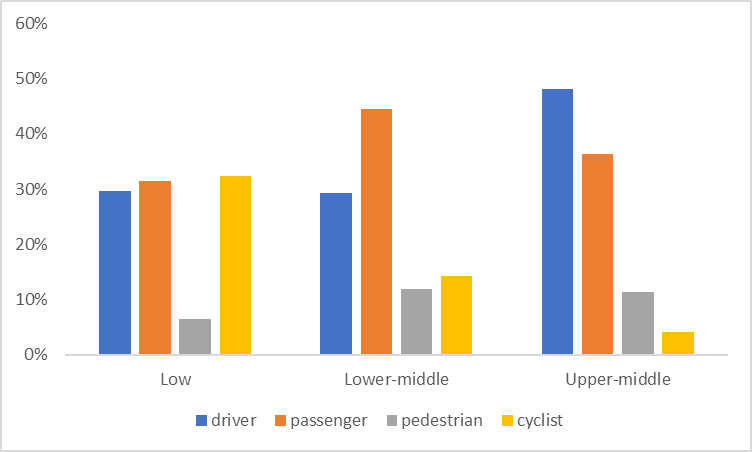


Figure 5A. Road user by World Bank income group

**
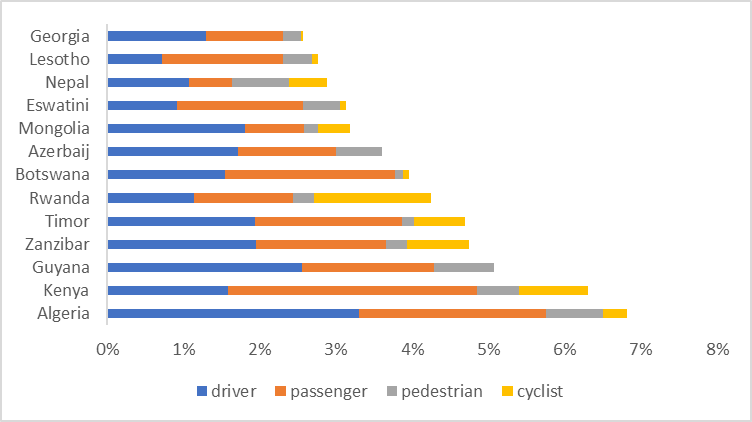
**

Figure 5B. Road user by country; Azerbaijan and Guyana did not ask about cyclists as road users.


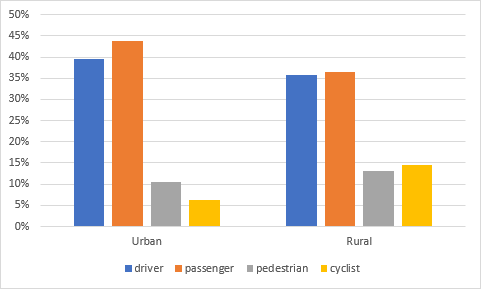


Figure 5C. Road user by geographical location; Azerbaijan and Guyana did not ask about cyclists as road users.
