# Supplementary material for: Individual characteristics associated with road traffic collisions and healthcare seeking in low- and middle-income countries and territories
Source: PLOS Glob Public Health. 2024 Jan 19;4(1):e0002768. doi: 10.1371/journal.pgph.0002768 (PMC10798533; doi:10.1371/journal.pgph.0002768)
Supplement: S6 Text — (DOCX) [file pgph.0002768.s006.docx]

**S6.**

Associations with occurrence of a non-fatal RTC in the previous 12 months for individual countries. Brazil and Ghana did not have information on non-fatal RTC incidence.

|  | OR | (95% CI) | |  |  |
| --- | --- | --- | --- | --- | --- |
| **Algeria**  **RTA%: 6.8**  **95%CI: 5.9 – 7.9** |  |  |  |  |  |
| Age | 0.99 | (0.98, 1.0) | |  |  |
| Sex | 0.38 | (0.29, 0.48) | |  |  |
| Married | 0.76 | (0.57, 1.01) | |  |  |
| Education | 1.0 (Ref) |  |  |  |  |
| Completed Primary | 1.17 | (0.87, 1.56) | |  |  |
| Some High School | 0.86 | (0.63, 1.18) | |  |  |
| High School or above | 1.29 | (0.88, 1.91) | |  |  |
|  |  |  |  |  |  |
| **Azerbaijan**  **RTA%: 3.6**  **95%CI: 2.6 – 4.9** |  |  |  |  |  |
| Age | 0.98 | (0.96, 1.01) | |  |  |
| Sex | 0.43 | (0.25, 0.72) | |  |  |
| Married | 1.36 | (0.66, 2.81) | |  |  |
| Education | 1.0 (Ref) |  |  |  |  |
| Completed Primary | 1. |  |  |  |  |
| Some High School | 1.38 | (0.75, 2.52) | |  |  |
| High School or above | 1. |  |  |  |  |
|  |  |  |  |  |  |
| **Botswana**  **RTA%: 4.0**  **95%CI: 3.1 – 5.1** |  |  |  |  |  |
| Age | 1.02 | (0.99, 1.06) | |  |  |
| Sex | 0.68 | (0.41, 1.14) | |  |  |
| Married | 0.92 | (0.53, 1.60) | |  |  |
| Education | 1.0 (Ref) |  |  |  |  |
| Completed Primary | 2.43 | (1.02, 5.81) | |  |  |
| Some High School | 1.37 | (0.53, 3.50) | |  |  |
| High School or above | 3.71 | (1.36, 10.17) | |  |  |
|  |  |  |  |  |  |
| **Eswatini**  **RTA%: 3.1**  **95%CI: 2.2 – 4.3** |  |  |  |  |  |
| Age | 0.98 | (0.94, 1.01) | |  |  |
| Sex | 0.47 | (0.24, 0.90) | |  |  |
| Married | 1.03 | (0.51, 2.11) | |  |  |
| Education | 1.0 (Ref) |  |  |  |  |
| Completed Primary | 0.94 | (0.37, 2.39) | |  |  |
| Some High School | 1.01 | (0.43, 2.38) | |  |  |
| High School or above | 1.54 | (0.63, 3.78) |  |  |  |
|  |  |  |  |  |  |
| **Georgia**  **RTA%: 2.6**  **95%CI: 1.9 – 3.4** |  |  |  |  |  |
| Age | 0.96 | (0.94, 0.98) |  |  |  |
| Sex | 0.38 | (0.22, 0.66) | |  |  |
| Married | 0.72 | (0.37, 1.40) | |  |  |
| Education | 1.0 (Ref) |  | |  | |
| Completed Primary | 1.54 | (0.34, 7.08) |  |  |  |
| Some High School | 0.99 | (0.39, 2.47) | |  |  |
| High School or above | 1. |  | |  |  |
|  |  |  |  |  |  |
| **Guyana**  **RTA%: 5.1**  **95%CI: 4.0 – 6.4** |  |  |  |  |  |
| Age | 1. | (0.98, 1.02) | |  |  |
| Sex | 0.47 | (0.28, 0.81) | |  |  |
| Married | 0.81 | (0.49, 1.34) | |  |  |
| Education | 1.0 (Ref) |  |  |  |  |
| Completed Primary | 2.59 | (0.77, 8.66) | |  |  |
| Some High School | 2.5 | (0.74, 8.44) | |  |  |
| High School or above | 4.67 | (1.24, 17.53) | |  |  |
|  |  |  |  |  |  |
| **Kenya**  **RTA%: 6.3**  **95%CI: 4.9 – 8.2** |  |  |  |  |  |
| Age | 0.99 | (0.97, 1.0) | |  |  |
| Sex | 0.41 | (0.28, 0.59) | |  |  |
| Married | 0.63 | (0.39, 1.03) | |  |  |
| Education | 1.0 (Ref) |  |  |  |  |
| Completed Primary | 1.87 | (1.17, 2.99) | |  |  |
| Some High School | 1.8 | (0.82, 3.95) | |  |  |
| High School or above | 1.65 | (1.10, 2.46) | |  |  |
|  |  |  |  |  |  |
| **Lesotho**  **RTA%: 2.8**  **95%CI: 1.8 – 4.2** |  |  |  |  |  |
| Age | 0.99 | (0.97, 1.02) | |  |  |
| Sex | 0.29 | (0.13, 0.68) | |  |  |
| Married | 0.94 | (0.50, 1.76) | |  |  |
| Education | 1.0 (Ref) |  |  |  |  |
| Completed Primary | 1.61 | (0.58, 4.46) | |  |  |
| Some High School | 3.1 | (1.06, 9.05) | |  |  |
| High School or above | 5.25 | (1.95, 14.10) | |  |  |
|  |  |  |  |  |  |
| **Mongolia**  **RTA%: 3.2**  **95%CI: 2.5 – 4.1** |  |  |  |  |  |
| Age | 0.96 | (0.94, 0.98) | |  |  |
| Sex | 0.3 | (0.18, 0.51) | |  |  |
| Married | 0.77 | (0.45, 1.31) | |  |  |
| Education | 1.0 (Ref) |  |  |  |  |
| Completed Primary | 1.89 | (0.43, 8.28) | |  |  |
| Some High School | 3.76 | (1.01, 13.93) | |  |  |
| High School or above | 4.33 | (1.26, 14.84) | |  |  |
|  |  |  |  |  |  |
| **Nepal**  **RTA%: 2.9**  **95%CI: 2.1 – 3.9** |  |  |  |  |  |
| Age | 0.99 | (0.97, 1.01) | |  |  |
| Sex | 0.42 | (0.24, 0.73) | |  |  |
| Married | 1.47 | (0.56, 3.90) | |  |  |
| Education | 1.0 (Ref) |  |  |  |  |
| Completed Primary | 0.98 | (0.42, 2.26) | |  |  |
| Some High School | 0.88 | (0.35, 2.20) | |  |  |
| High School or above | 1.35 | (0.57, 3.17) | |  |  |
|  |  |  |  |  |  |
| **Rwanda**  **RTA%: 4.3**  **95%CI: 3.6 – 5.0** |  |  |  |  |  |
| Age | 0.97 | (0.95, 0.98) | |  |  |
| Sex | 0.28 | (0.20, 0.39) | |  |  |
| Married | 0.88 | (0.63, 1.23) | |  |  |
| Education | 1.0 (Ref) |  |  |  |  |
| Completed Primary | 1.3 | (0.93, 1.82) | |  |  |
| Some High School | 1.73 | (1.10, 2.74) | |  |  |
| High School or above | 1.61 | (0.55, 4.67) | |  |  |
|  |  |  |  |  |  |
| **Timor L’este**  **RTA%: 4.7**  **95%CI: 3.6 – 6.0** |  |  |  |  |  |
| Age | 1.01 | (0.98, 1.03) | |  |  |
| Sex | 0.32 | (0.20, 0.54) | |  |  |
| Married | 1.05 | (0.59, 1.86) | |  |  |
| Education | 1.0 (Ref) |  |  |  |  |
| Completed Primary | 0.75 | (0.36, 1.55) | |  |  |
| Some High School | 1.56 | (0.81, 3.01) | |  |  |
| High School or above | 1.73 | (0.78, 3.83) | |  |  |
|  |  |  |  |  |  |
| **Zanzibar**  **RTA%: 4.7**  **95%CI: 3.8 – 5.9** |  |  |  |  |  |
| Age | 0.97 | (0.95, 1.0) | |  |  |
| Sex | 0.26 | (0.14, 0.49) | |  |  |
| Married | 2.04 | (1.02, 4.07) | |  |  |
| Education | 1.0 (Ref) |  |  |  |  |
| Completed Primary | 1.54 | (0.71, 3.34) | |  |  |
| Some High School | 1.33 | (0.60, 2.93) | |  |  |
| High School or above | 1.52 | (0.54, 4.25) | |  |  |
