# Supplementary material for: Individual characteristics associated with road traffic collisions and healthcare seeking in low- and middle-income countries and territories
Source: PLOS Glob Public Health. 2024 Jan 19;4(1):e0002768. doi: 10.1371/journal.pgph.0002768 (PMC10798533; doi:10.1371/journal.pgph.0002768)
Supplement: S7 Text — (DOCX) [file pgph.0002768.s007.docx]

**S7**

Results of binary logistic analyses ascertaining the associations with non-fatal RTC and age, sex, marital and education status, and alcohol use in the past month in participants aged 25-64 years and 18-64 years.

|  | **Association of age, sex, marital and education status, and alcohol consumption on RTC in the previous month (age category 25-64 year, 10 countries, 32,476* participants)** | | | **Association of age, sex, marital and education status, and alcohol consumption on RTC in the previous month (age category 18-64 years; 8 countries 33,292** * **participants)** | | |
| --- | --- | --- | --- | --- | --- | --- |
|  | OR | 95% CI | P value | OR | 95% CI | P value |
| **Age** | 0.99 | 0.98-1.0 | 0.006 | 0.99 | 0.98-1.0 | 0.016 |
| **Sex (female)** | 0.38 | 0.30-0.48 | <0.001 | 0.40 | 0.33-0.49 | <0.001 |
| **Married or cohabiting (single)** | 0.67 | 0.52-0.87 | 0.003 | 0.75 | 0.61-0.93 | 0.009 |
| **Education (no education or less than primary)** |  |  |  |  |  |  |
| **Completed Primary** | 1.52 | 1.13-2.03 | 0.006 | 1.43 | 1.06-1.93 | 0.018 |
| **Some secondary** | 1.26 | 0.94-1.68 | 0.120 | 1.16 | 0.89-1.50 | 0.280 |
| **Completed secondary or more** | 1.62 | 1.26-2.08 | <0.001 | 1.52 | 1.19-1.94 | 0.001 |
| **Alcohol in last month** | 1.13 | 0.89-1.44 | 0.330 | 1.27 | 1.00-1.61 | 0.048 |

*Numbers in the multivariable analyses are lower than those used in the main descriptive analyses, given missingness of some variables.
