# Supplementary material for: Individual characteristics associated with road traffic collisions and healthcare seeking in low- and middle-income countries and territories
Source: PLOS Glob Public Health. 2024 Jan 19;4(1):e0002768. doi: 10.1371/journal.pgph.0002768 (PMC10798533; doi:10.1371/journal.pgph.0002768)
Supplement: S8 Text — (DOCX) [file pgph.0002768.s008.docx]

**S8**

Results of binary logistic analyses ascertaining the associations of age, sex, marital and education status, and wealth in participants aged 25-64 years and 18-64 years.

Only five countries and territories (13,042 participants) had data available on age, sex, marital and education status, and wealth for the age group 24-64 and three countries (12,412 participants) in the age group 18-64. Increasing wealth was associated with increasing odds of suffering an RTC (OR: 4.14, 95%CI: 2.01 – 8.57, P value: <0.001) for the highest compared with the lowest wealth quintile in participants aged 25-65 and (OR: 4.52, 95%CI 2.09 – 9.78, P value <0.001) for the highest compared to the lowest wealth quintile in participants aged 18-64). Age remained negatively associated with suffering an RTC, RTC was also significantly lower in females compared with males, and with those who were married or cohabiting vs single in the older age group. in both age samples, the associations between RTC and education became statistically insignificant.

|  | **age category 25-64 (analysis in 5 countries with 15,042 * participants)** | | | **age category 18-64 (analysis in 3 countries with 12,412* participants)** | | |
| --- | --- | --- | --- | --- | --- | --- |
|  | OR | 95% CI | P value | OR | 95% CI | P value |
| **Age** | 0.98 | 0.97-1.00 | 0.045 | 0.99 | 0.98-1.00 | 0.200 |
| **Sex (female)** | 0.40 | 0.28-0.55 | <0.001 | 0.38 | 0.28-0.51 | <0.001 |
| **Married or cohabiting (single)** | 0.63 | 0.40-1.00 | 0.048 | 0.90 | 0.61-1.34 | 0.609 |
| **Education (no education or less than primary)** |  |  |  |  |  |  |
| **Completed Primary** | 1.51 | 0.99-2.30 | 0.055 | 1.17 | 0.76-1.80 | 0.482 |
| **Some secondary** | 1.23 | 0.60-2.53 | 0.569 | 0.78 | 0.39-1.56 | 0.485 |
| **Completed secondary or more** | 0.94 | 0.64-1.36 | 0.737 | 0.78 | 0.52-1.17 | 0.221 |
| **Wealth (Q1)** |  |  |  |  |  |  |
| **Q2** | 1.39 | 0.81-2.41 | 0.234 | 2.08 | 1.13-3.85 | 0.019 |
| **Q3** | 2.99 | 1.56-5.75 | 0.001 | 4.19 | 2.23-7.85 | <0.001 |
| **Q4** | 2.17 | 1.22-3.85 | 0.008 | 2.49 | 1.34-4.60 | 0.004 |
| **Q5** | 4.14 | 2.01-8.57 | <0.001 | 4.52 | 2.09-9.78 | <0.001 |

*Numbers in the multivariable analyses are lower than those used in the main descriptive analyses, given missingness of some variables.
