# Supplementary material for: Individual characteristics associated with road traffic collisions and healthcare seeking in low- and middle-income countries and territories
Source: PLOS Glob Public Health. 2024 Jan 19;4(1):e0002768. doi: 10.1371/journal.pgph.0002768 (PMC10798533; doi:10.1371/journal.pgph.0002768)
Supplement: S9 Text — (DOCX) [file pgph.0002768.s009.docx]

**S9.**

Associations of rural or urban habitation on suffering an RTC

The models including rural vs. urban household residency in the 7 countries and territories and 25,946 participants between 25-64 years and 6 countries and 27,111 participants between 18-64 years suggests no association between rural or urban status and suffering an RTC.

Associations of rural or urban habitation on suffering an RTC

|  | **age category 25-64 (analysis in 7 countries with 25,946* participants)** | | | **age category 18-64 (analysis in 6 countries with 27,111 * participants)** | | |
| --- | --- | --- | --- | --- | --- | --- |
|  | **OR** | **95% CI** | **P value** | **OR** | **95% CI** | **P value** |
| **Age** | 0.99 | 0.98-0.99 | 0.003 | 0.99 | 0.98-1.00 | 0.007 |
| **Sex (female)** | 0.39 | 0.32-0.47 | <0.001 | 0.41 | 0.34-0.48 | <0.001 |
| **Being Married** | 0.71 | 0.55-0.92 | 0.009 | 0.79 | 0.64-0.98 | 0.034 |
| **Education (no education or less than primary)** |  |  |  |  |  |  |
| **Completed Primary** | 1.40 | 1.08-1.81 | 0.011 | 1.37 | 1.04-1.80 | 0.027 |
| **Some secondary** | 0.97 | 0.71-1.31 | 0.834 | 0.93 | 0.70-1.23 | 0.591 |
| **Completed secondary or more** | 1.09 | 0.86-1.39 | 0.471 | 1.14 | 0.89-1.46 | 0.313 |
| **Geography (Rural)** | 0.63 | 0.48-0.81 | 0.001 | 0.71 | 0.55-0.92 | 0.009 |

*Numbers in the multivariable analyses are lower than those used in the main descriptive analyses, given missingness of some variables.
