# Supplementary material for: Individual characteristics associated with road traffic collisions and healthcare seeking in low- and middle-income countries and territories
Source: PLOS Glob Public Health. 2024 Jan 19;4(1):e0002768. doi: 10.1371/journal.pgph.0002768 (PMC10798533; doi:10.1371/journal.pgph.0002768)
Supplement: S10 Text — (DOCX) [file pgph.0002768.s010.docx]

**S10**

Multivariable analysis of the associations of requiring medical attention after an RTC in the previous 12 months with age, sex, marital status, and wealth.

|  | **age category 25-64 (analysis in 3 countries with 7,982 participants)** | | |
| --- | --- | --- | --- |
|  | OR | 95% CI | P value |
| **Age** | 1.00 | 0.97-1.02 | 0.667 |
| **Sex (female)** | 0.29 | 0.17-0.44 | <0.001 |
| **Married or cohabiting (single)** | 0.93 | 0.49-1.54 | 0.811 |
| **Wealth (Q1)** |  |  |  |
| **Q2** | 2.27 | 0.92-6.02 | 0.084 |
| **Q3** | 5.79 | 2.55-14.47 | <0.001 |
| **Q4** | 4.25 | 1.94-12.54 | 0.002 |
| **Q5** | 3.50 | 2.04-13.03 | 0.009 |
